# Pulsed field ablation in patients with cardiac implantable electronic devices: an ex vivo assessment of safety

**DOI:** 10.1007/s10840-024-01758-2

**Published:** 2024-05-22

**Authors:** Carsten Lennerz, Matthew O’Connor, Claudia Schaarschmidt, Tilko Reents, Felix Bourier, Marta Telishevska, Sarah Lengauer, Miruna Popa, Katharina Wimbauer, Ellen Holmgren, Mara Thoma, Lovis Spitzauer, Fabian Bahlke, Hannah Krafft, Florian Englert, Katharina Knoll, Lena Friedrich, Patrick Blazek, Gabriele Hessling, Christof Kolb, Isabel Deisenhofer, Marc Kottmaier

**Affiliations:** 1 Department of Electrophysiology, German Heart Centre Munich, Technical University of Munich, Lazarettstr. 36, 80636 Munich, Germany; 2 DZHK (German Centre for Cardiovascular Research), partner site Munich Heart Alliance, Munich, Germany; 3 Cardiology Department, Auckland City Hospital, Auckland, New Zealand

**Keywords:** Electromagnetic interference, Pulsed field ablation, Atrial fibrillation ablation, Pacemaker, Implantable cardioverter-defibrillator

## Abstract

**Background:**

Pulse field ablation (PFA) is a novel catheter ablation technology with potential safety benefits due to its tissue selectivity. It has the potential to directly damage or interact with the functionality of cardiac implantable electronic devices (CIEDs) in the form of electromagnetic interference (EMI). The aim of our study was to assess the impact of PFA on CIEDs.

**Methods:**

PFA lesions (45 per CIED) were applied from the Farapulse system to CIEDs (< 5 cm from the lead tip and < 15 cm from the generator). All devices were checked before and after PFA application for proper sensing and pacing functionality as well as for integrity of shock circuits in ICDs using a heart simulator. Moreover, devices were then interrogated for any spontaneous reprogramming, mode switching or other EMI effects.

**Results:**

In total, 44 CIEDs were tested (16 pacemaker, 21 ICDs, 7 CRT-P/D) with 1980 PFA applications. There was no change in device settings, functionality and electrical parameters, and there was no macroscopic damage to the devices. The risk of damage to the electric components or leads on a patient-based analysis is 0/44 (95% CI 0–8%) and on a PFA pulse-based analysis is 0/1980 (95% CI 0–0.2%). Clinically relevant EMI appeared with oversensing and pacing inhibition but not tachycardia detection.

**Conclusions:**

Bipolar PFA appears safe and does not result in damage to CIEDs or leads. Clinically relevant EMI does occur, but appropriate peri-procedural programming may mitigate this. *In vivo* studies are needed to confirm our findings.

**Graphical Abstract:**

**Figure:**
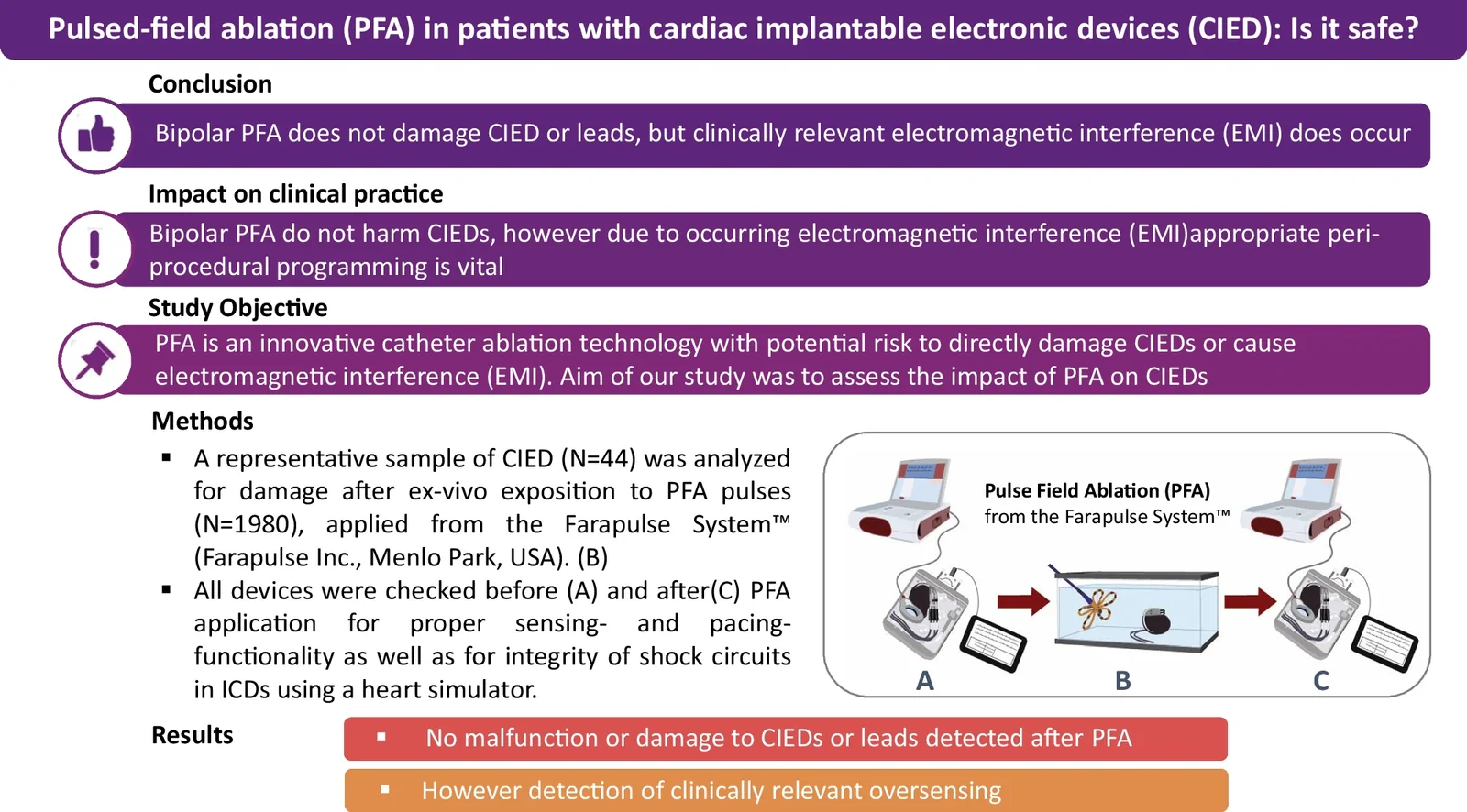

**Supplementary Information:**

The online version contains supplementary material available at https://doi.org/10.1007/s10840-024-01758-2.

## Introduction

Catheter ablation is a well-established therapy for management of atrial fibrillation (AF) [1]. Despite improvements in the safety of catheter ablation, there continues to be a risk of complication due to injury of adjacent structures such as the phrenic nerve and oesophagus [1–3]. The drive to reduce the risk associated with catheter ablation has resulted in the development of pulsed field ablation (PFA). PFA is a novel non-thermal energy source that leads to irreversible electroporation by induction of intense but short-duration electrical fields which leads to an electropermeabilization in myocardial cell membranes, resulting in cell death [4]. Reportedly, different cell types have a different sensitivity to PFA resulting in myocardial selectivity of PFA and the avoidance of collateral injury [5, 6]. Recent trials have demonstrated that PFA holds great promise in the field of AF ablation, not only in the rapidity of the ablation but also in reducing the risk of phrenic nerve or oesophageal injury [7–10].

PFA generates an electromagnetic field during ablation, and there is no data on its strength or effect on cardiac implantable electronic devices (CIEDs). There is the potential for electromagnetic interference (EMI) from PFA to affect the functionality of a CIED. Therefore, the presence of a CIED was an exclusion criterion in the large PFA-randomized trials like PEFCAT and IMPULSE [10]. The electroporation voltage (up to 2000 V) could potentially lead to changes of CIED functionality or permanent damage to the electronic components. In particular, there is a risk of oversensing resulting in pacing inhibition or inappropriate therapy (ICDs), mode switching, spontaneous mode switching or power on reset.

The aim of this trial was to assess the impact of PFA using the Farapulse System™ on CIEDs.

## Methods

### Model

**Fig. 1:**
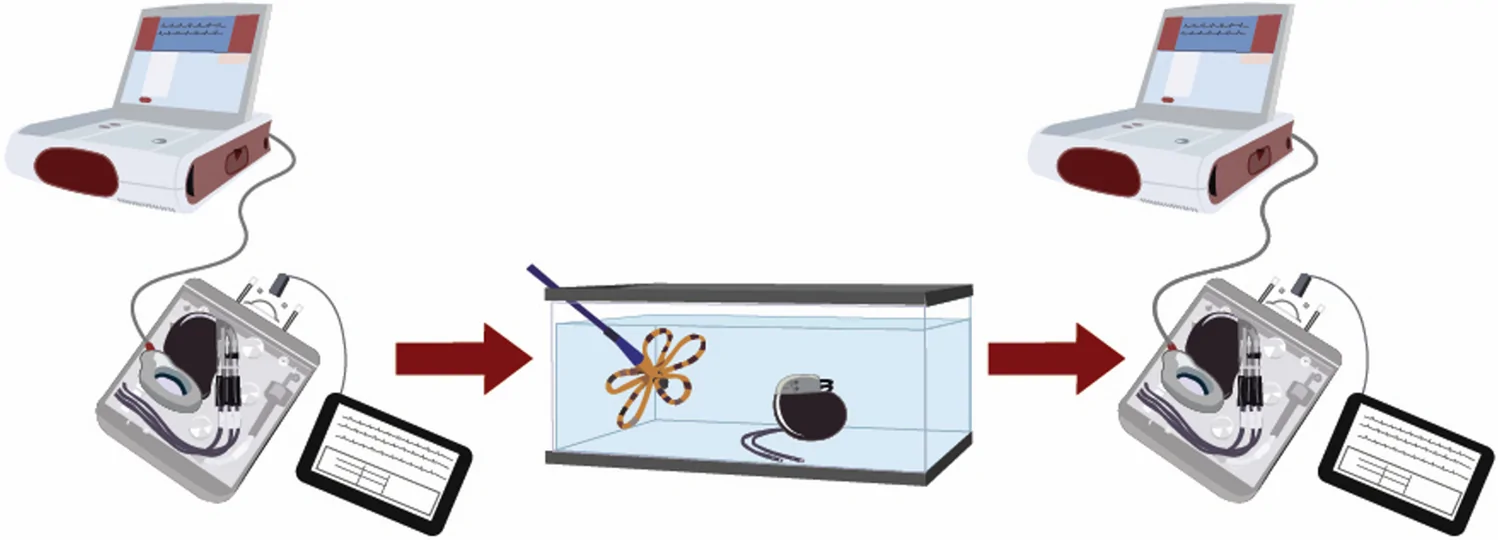
Illustration of the investigational model used. CIED functionality is evaluated with the use of a heart simulator. The CIED is then connected to leads, placed in a saline-filled container (temperature 37 °C, electric impedance 120–130 Ω) and the PFA catheter placed in close proximity to CIED generator (< 15 cm) and lead tip (< 5 cm) before PFA is applied. The CIED is monitored throughout with device telemetry when available and then functionality is re-tested after PFA

CIEDs were obtained from recent interventions (such as upgrade procedures or system removal due to CIED associated infections). Devices explanted due to having reached ERI were excluded as device interrogation can be limited at ERI and this state is less clinically relevant (ablations are seldom performed on a patient with a CIED at ERI). All CIEDs were in fully functioning condition with a battery longevity of > 1 year. Each CIED was, in turn, connected to a heart simulator (InterSim III Touch, TQ-Systems GmbH, Seefeld, Germany), and baseline CIED functionality was tested. Devices were then connected to leads and placed in a saline bath for PFA application. The saline bath temperature was continuously kept at 37 °C, and the electric impedance was maintained at 120–130 Ω by adjusting the ratio of saline and water (Fig. 1). Testing included measurement of sensing values, defined pacing thresholds, impedance values, battery status and programmed settings. In ICDs, the parameters for the high-voltage circuits, shock impedance, charge time and energy delivered were measured.

### PFA application

For PFA, the Farapulse System™ with the over-the-wire Farawave™ catheter (Farapulse Inc., Menlo Park, CA, USA) was used. The PFA generator’s ablation waveform was limited in each patient to programmed pulse parameters and was not modifiable by the investigator. The modifiable generator output was set to the maximum level of 2000 V (biphasic) with each application consisting of five microsecond-scale biphasic pulses with 2000 V in a bipolar fashion.

The CIED was connected to leads which were positioned in the saline bath, and the PFA catheter was positioned < 5cm from the lead tip and < 15cm from the generator to model the real-life proximity likely to be encountered with cardiac ablation with PFA (Fig. 1). PFA was then applied for a total of 45 ‘lesions’ before the CIED was re-interrogated with use of the heart simulator. The 45 lesions were chosen as it takes approximately eight lesions to isolate a pulmonary vein (32 lesions for bilateral PVI) with an additional 13 lesions to account for potential ‘PVI-plus’ ablation approaches (left or right atrial linear ablation or posterior wall ablation). In two devices, real-time intracardiac electrograms were collected during PFA application via device telemetry.

### CIED interrogation and testing

Both before and after PFA application, devices were tested for battery status, sensing and pacing parameters and impendence measurements (measurements recorded are the mean of three tests). Subsequent to PFA application, devices were interrogated to check for spontaneous reprogramming or power-on reset. In order to test the sensing, the heart simulator was programmed to deliver atrial signals with 1.5 mV and 2.5 mV and right or left ventricle signals with a amplitude of 6.0 mV or 12.5 mV, respectively. Pacing output was tested at two outputs: 1 V @ 0.4 ms and maximum output with maximal pulse width. The programmed output was then compared to the voltage detected by the heart simulator.

In all high-voltage devices, a shock was applied before and after PFA, and the relevant shock parameters, including shock impedance, charge time and delivered shock energy, were evaluated. In all ICD systems, ventricular fibrillation (VF) was simulated via heart simulator, and CIED detection was evaluated.

### Statistics

Continuous variables are presented as mean +/− standard deviation or median. Categorical data are expressed as frequencies and percentages. Univariate comparisons were performed using a paired *t* test. A *p*-value of < 0.05 was considered statistically significant. All analyses were performed using SPSS for Mac version 25.0 (SPSS Inc., Chicago, IL, USA).

## Results

In total, 44 CIED were tested (16 pacemaker, 21 ICDs, 7 CRT-P/D) with 1980 pulse field ablations (45 ablations per device). A wide spectrum of CIEDs was represented in the study (Table 1).

**Table 1:** Cardiac implantable electronic devices CIEDs tested for electromagnetic interference (EMI) and damage due to pulse field ablation (PFA)

Manufacturer	Device	Type of device	Number of devices
Abbott	Endurity Core SR	VVI-PM	2
	Endurity Core DR	DDD-PM	1
	Fortify Assura VR	VVI-ICD	2
	Fortify Assura DR	DDD-ICD	1
	Quadra Assura	CRT-ICD	3
Biotronik	Ecuro DR	DDD-PM	1
	Enitra 6 DR	DDD-PM	5
	Inlexa 3 VR-T	VVI-ICD	3
	Intica 5 VR-T	VVI-ICD	1
	Iforia 5 VR-T DX	VDD-ICD	1
	Iforia 3 DR-T	DDD-ICD	1
	Intica 5 DR-T	DDD-ICD	1
	Intica 5 HF-T	CRT-ICD	1
Boston Scientific	Charisma EL SR	VVI-ICD	1
	Dynagen VR	VVI-ICD	2
	Inogen VR	VVI-ICD	1
	Charisma EL DR	DDD-ICD	1
	Inogen Mini DR	DDD-ICD	1
	Inogen CRT-D	CRT-ICD	1
Medtronic	Astra XT DR MRI	DDD-PM	1
	Ensura DR MRI	DDD-PM	2
	Solara MRI CRT-P	CRT-PM	1
	Evera MRI S VR	VVI-ICD	1
	Evera MRI S DR	DDD-ICD	3
	Amplia MRI CRT-D	CRT-ICD	1
Microport	Teo SR	VVI-PM	1
	Teo DR	DDD-PM	1
	Kora 250 DR	DDD-PM	1
	Platinium VR	VVI-ICD	1
Vitatron	Q20 SR	VVV-PM	1

*ICD* implantable cardioverter defibrillator, *PM* pacemaker, *VVI* single camber device, *DDD* dual chamber device, *CRT* cardiac resynchronization therapy device

### Acute EMI

**Fig. 2:**
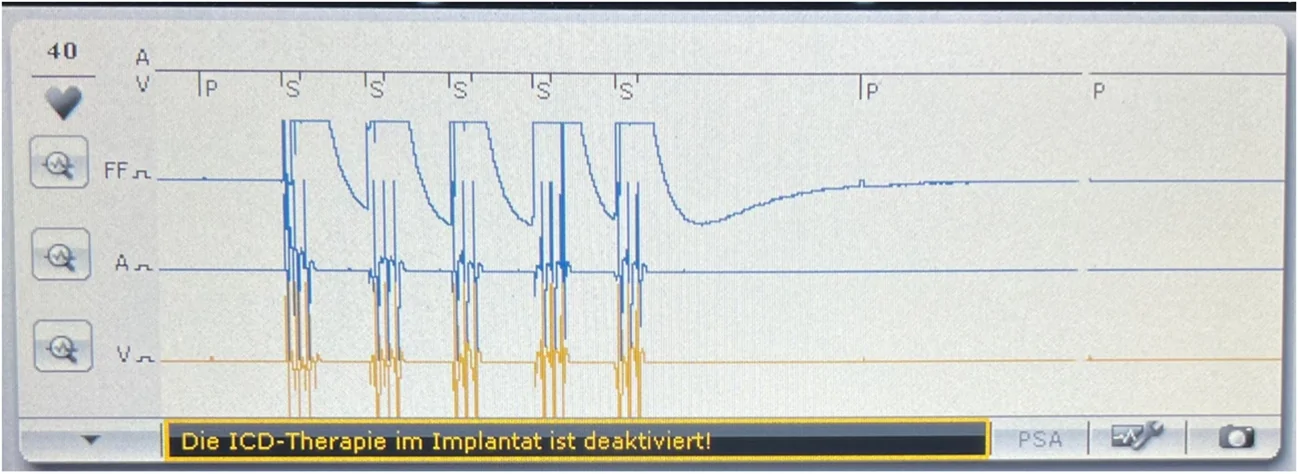
Real-time electrograms from a dual chamber ICD during PFA application. Over-sensing is demonstrated by the sense marker (‘S’) and results in pacing inhibition during PFA. Pacing inhibition resulted in a 6-s pause in ventricular pacing

There were no incidences of triggering of tachycardia detection, mode switching or noise detection alerts. Though our study was not designed to investigate the incidence of pacing inhibition, we undertook continuous telemetric monitoring in two cases and observed over-sensing during PFA application. There was a single sensed event for each pulse delivered resulting in five sensed events that resulted in pacing inhibition (Fig. 2 and Supplementary Video 1).

### CIED integrity

**Fig. 3:**
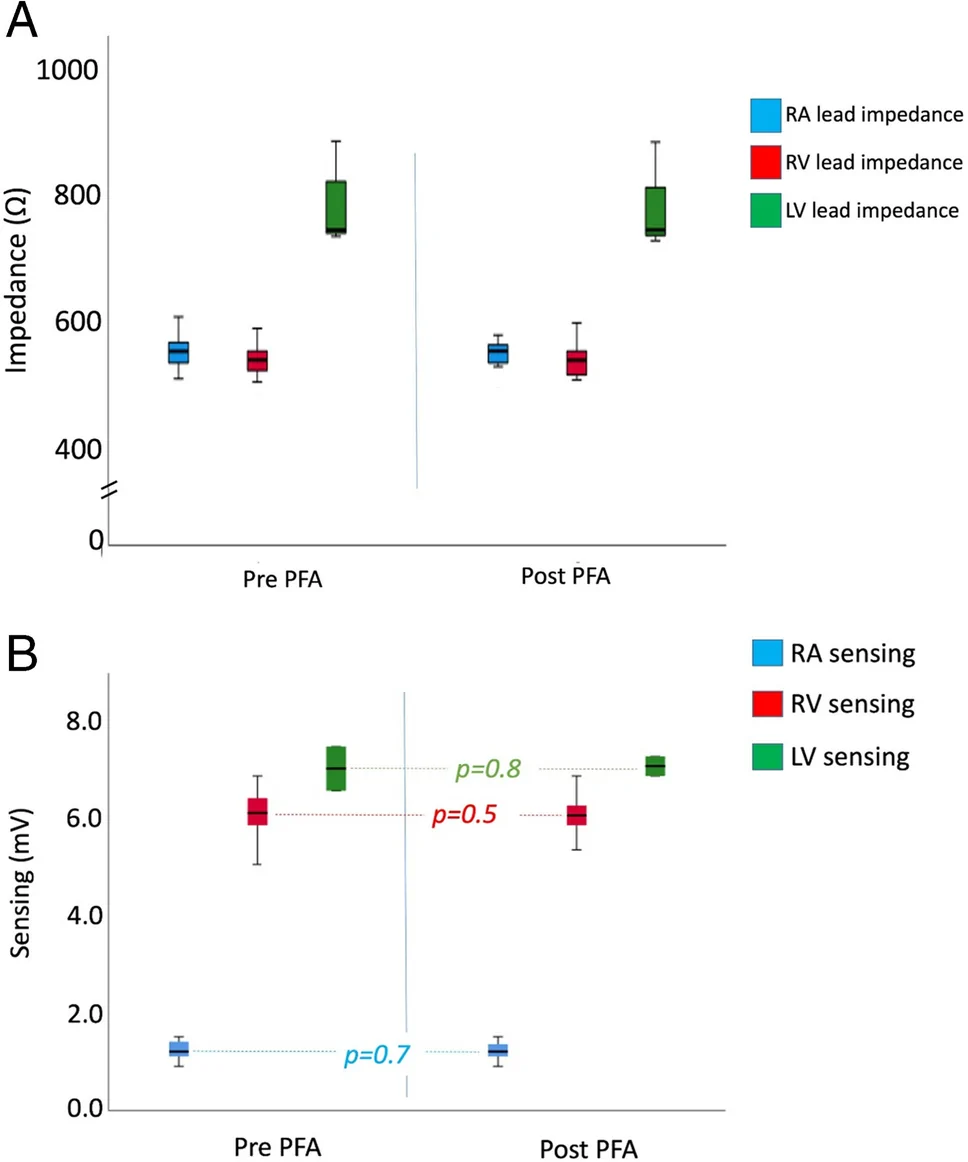
**A** Values for lead impedance measurement pre- (1) and post-PFA application (2). **B** Sensing values pre- and post-PFA application

PFA application did not result in changes in device settings, and there was no evidence of malfunction or visual damage to the CIED generator or leads. Battery status was unchanged post-PFA applications (mean battery voltage 2.9+/−0.09 V vs 2.9+/−0.1 V, *p* = 0.5). PFA application did not result in changes in lead impedance: mean RA lead impedance 552.08+/−26.23 Ω vs 550.37+/−26.88 Ω (*p* = 0.82), mean RV lead impedance 538.14+/−26.33 Ω vs 537.19+/−29.47 Ω (*p* = 0.87) and mean LV lead impedance 745.90+/−121.22 Ω vs 743.90+/−115.15 Ω (*p* = 0.98) (Fig. 3A).

### Sensing and pacing

PFA applications did not lead to changes in sensing of the CIEDs. There was no significant difference in sensed values: mean RA sensing 1.37+/−0.80 mV vs 1.36+/−0.87 mV (*p* = 0.7), mean RV sensing 6.02+/−0.6 mV vs 6.00+/−0.60 mV (*p* = 0.50) and mean LV sensing 7.00+/−0.28 mV vs 7.00+/−0.6 mV (*p* = 0.80) (Fig. 3B).

**Fig. 4:**
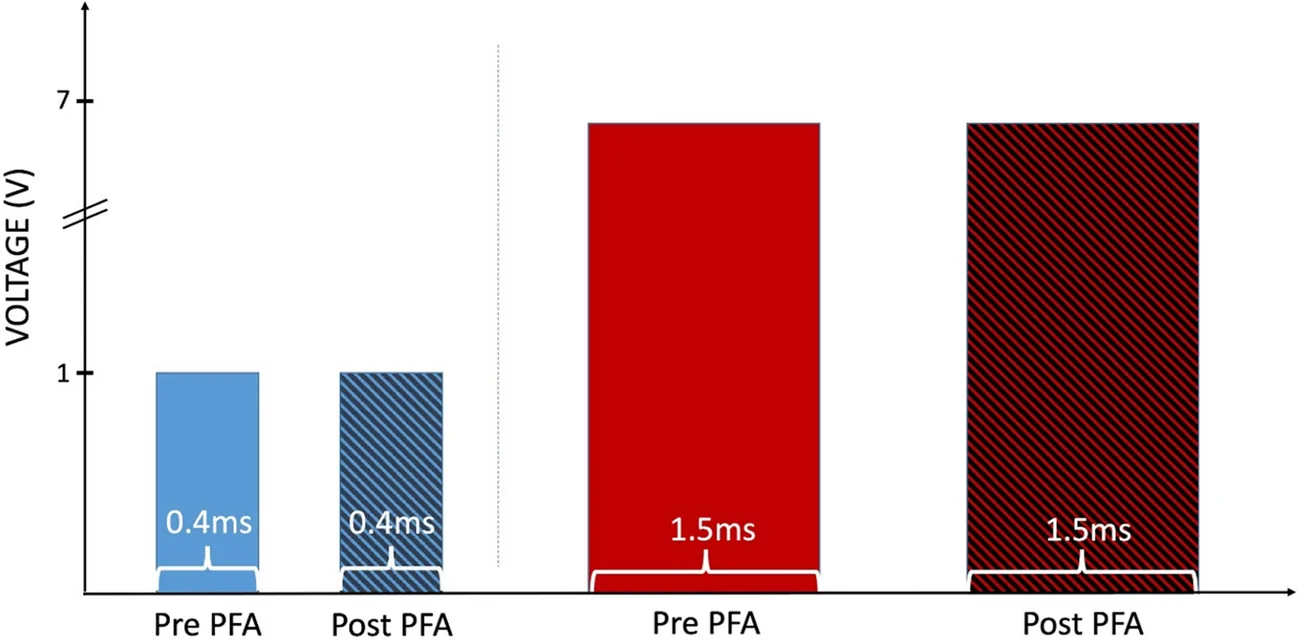
Measured pacing output pre-PFA and post-PFA application. Pacing was programmed either to the nominal value of 1 V @ 0.4 ms (blue) or to the maximal programmable values (red)

Finally, device pacing output at both nominal (1.0 V @ 0.4 ms) and the highest output/longest pulse width did not change post-PFA application (Fig. 4).

### Anti-tachycardia therapy

PFA applications did not alter anti-tachycardia therapy capability of ICD and CRT-Ds. All devices were able to detect ventricular fibrillation appropriately without dropout post-PFA application. Mean delivered shock energy was the same post-PFA application (38.80+/−2.30 J vs 38.80+/−2.30 J, *p* = 0.57); mean charging time was also unchanged after PFA application (9.7+/−1.56 s vs 9.80+/−1.21 s, *p* = 0.84) (Supplementary Video 2). Finally, RV Shock impedance was unchanged post-PFA (71.44+/−16.11 Ω vs 71.72+/−16.36 Ω, *p* = 0.51).

Overall, the risk of damage to the electric components or leads on a patient-based analysis is 0/44 (95% CI 0–8%), and the risk of such damage on a PFA pulse-based analysis is 0/1980 (95% CI 0–0.2%).

## Discussion

Pulse field ablation is a promising new technology in interventional electrophysiology. The reported tissue specificity has significant appeal regarding the safety of AF ablation procedures, minimizing the risk of oesophageal, phrenic nerve and coronary artery damage. As the indication and clinical application of catheter ablation expands, the number of patients with a CIED who may benefit from catheter ablation with PFA will continue to increase. The uptake of PFA as a technology will be limited if patients with CIEDs are precluded from its use. Furthermore, patients with CIEDs may be denied potentially safer procedures.

In our study, we observed no damage to the electric components of the CIED or to the leads during multiple PFA applications suggesting that there are no long-term safety concerns applicable to the use of PFA in patients with CIEDs. We did however observe clinically meaningful EMI manifesting as over-sensing and subsequent pacing inhibition. We observed that each PFA pulse resulted in a sensed event; when considering a pacing rate of 40 bpm and five PFA pulses per lesion, there would be three inhibited paced beats potentially resulting in a six second pause. Due to the frequency of the five pulses (microsecond scale), the PFA application duration is too short for triggering ATP in ICDs.

A study on the use of electrochemotherapy/electroporation of the liver in patients with CIEDs also demonstrated EMI in the form of pacing inhibition during pulse application complimenting our data suggesting that EMI may be a clinical feature of PFA [11]. Two previous studies evaluated a total of 21 patients with CIEDs (15 pacemakers, 3 ICDs and 3 CRT-Ds in total) undergoing PFA for atrial fibrillation [12, 13]. In these patients, there was no reported adverse outcomes or EMI; it is possible that the *in vivo* distance from the PFA catheter to the CIED components was greater than in our study explaining difference in results. With anatomical variation, the *in vivo* distances may be shorter, and EMI may occur such as in our study. Given the short duration of PFA, the clinical sequalae (such as inhibition of pacing) are likely minimal.

Our results further add to the growing body of literature of relative safety of PFA in patients with CIEDs. Additionally, our study is the first to actively test the high voltage components of ICDs after PFA application.

The optimal PFA delivery settings are not yet known and already there are variations between manufactures/systems. The variation in voltage, pulse duration, pulse frequency, number of pulses and the use of mono-polar PFA may all result in a different pattern of EMI. There is the potential for such settings to result in a situation where PFA-related EMI results in tachycardia detection or prolonged asystole in pacing dependent patients or potential damaging of the CIED.

Current recommendations already advocate for the use of temporary programming changes during MRI scans and surgery requiring electrocautery, common situations where EMI occurs [14]. We would advocate for a similar approach to CIED patients during PFA ablation to minimize the risk of clinical adverse events. We recommend considering the use of a RV catheter for back up pacing or asynchronous mode in pacing dependent patients. For patients who are not pacing dependent programming should be considered before undertaking PFA ablation and tailored to the clinical situation. Anti-tachycardia therapies should be disabled. Finally, all CIEDs should have a complete device interrogation prior to and after PFA ablation.

Our study was conducted in an ex vivo model and may not be representative of *in vivo* biophysics. We investigated PFA with a single, bipolar setting, and as such, our results may not be applicable to other potential PFA settings. For example, the electromagnetic field in monopolar PFA is potentially larger and may have a greater influence on CIEDs and warrants further study. Finally, we did not evaluate PFA applied directly onto leads as may occur with right atrial or right ventricular PFA use; therefore, we would currently advise against direct application of PFA onto leads or lead/myocardium interface. Finally, we did not evaluate the use of PFA in leadless pacemakers or sub-cutaneous ICDs, and our results cannot be generalized to this population.

## Conclusion

PFA using the Farapulse system appears to be safe and does not result in malfunction or damage to CIEDs or leads. We did observe clinically relevant EMI, but with appropriate peri-procedural programming, this can be mitigated. Larger, *in vivo* series are needed to confirm safety of PFA in patients with CIEDs. Different PFA application techniques such as monopolar PFA remain untested.

## Supplementary Information

Below is the link to the electronic supplementary material.

Supplementary file1 (MOV 39294 KB)

Supplementary file2 (MOV 110705 KB)

## Data Availability

The full dataset is available upon reasonable request.

## Abbreviations

AF: Atrial fibrillation
CIED: Cardiac implantable electronic device
CRT: Cardiac resynchronization therapy
EMI: Electromagnetic interference
ERI: Elective replacement indicator
ICD: Implantable cardioverter defibrillator
PFA: Pulsed field ablation
PVI: Pulmonary vein isolation
RV: Right ventricle
VF: Ventricular fibrillation
